# Human microglia development in the embryonic brain

**DOI:** 10.1093/lifemedi/lnac029

**Published:** 2022-08-10

**Authors:** Baoguo Li, Yanxin Li, Zhongqiu Li, Jianwei Jiao, Ido Amit

**Affiliations:** Department of Immunology, Weizmann Institute of Science, Rehovot 76100, Israel; State Key Laboratory of Stem Cell and Reproductive Biology, Institute of Zoology, Chinese Academy of Sciences, Beijing 100101, China; State Key Laboratory of Stem Cell and Reproductive Biology, Institute of Zoology, Chinese Academy of Sciences, Beijing 100101, China; State Key Laboratory of Stem Cell and Reproductive Biology, Institute of Zoology, Chinese Academy of Sciences, Beijing 100101, China; Department of Immunology, Weizmann Institute of Science, Rehovot 76100, Israel

Microglia play essential roles in human neural development. But the microglia development in the human fetal brain is still not well understood. Recently several studies have uncovered the development of human microglia at single-cell resolution. In this research highlight, we summarize the advance in human microglia development.

As the resident myeloid cells of the central nervous system (CNS), microglia play important roles in immunity, surveillance, and protection of the healthy CNS [1]. Emerging evidence has demonstrated that microglia derive from erythromyeloid progenitors in the yolk sac, which migrate, colonize, differentiate, and mature in the brain and eventually reach a steady state that maintains the immune balance of the CNS environment [2]. Compared with mature microglia, embryonic microglia show significant heterogeneity and participate in various physiological activities, including cell proliferation, migration, gliogenesis, angiogenesis, and synaptic construction.

The rapid development of single-cell omics technology has provided a powerful tool for understanding cell fate determination and cell heterogeneity. In recent years, using single-cell sequencing technology also accelerated our knowledge about microglia development in mice. Single-cell transcriptomic analysis with genome-wide chromatin and expression profiling identified three temporal microglia developmental stages-embryonic, early postnatal, and adult [2]. Subsequent studies provide a more comprehensive picture of microglia’s spatial and temporal heterogeneity during development, aging, and diseases [3, 4].

However, species differences between humans and mice significantly limit our understanding of human microglia differentiation and developmental function, especially during early embryonic development [5]. In addition, human single-cell sequencing research has been limited by technical limitation such as focus on nuclear sequencing and focus on early origin of microglia, from the yolk sac to the entry of the brain, or the development of microglia in the whole CNS. Additional knowledge is missing regarding regional specialization, fate determination, and state transition of microglia in the early development of the human fetal brain.

In one recent paper published in Cell Stem Cell [6], Li and colleagues studied the microglia in different regions during human brain development, and accurately revealed the spatiotemporal dynamic characteristics of microglia regional specialization and state transition in the developing human brain, and evaluated the conservation and molecular differences of state transition between the two species by comparing the differences of microglia transcriptome in mouse and human cortex. Using droplet based scRNA-seq, the authors profiled molecular changes from the yolk sac and head tissues at Carnegie Stage (CS) 12, as well as brain regions including cortex (Cor), diencephalon (Dien), midbrain (Mid), and cerebellum (Cere) at multiple gastrulation weeks (GW8, GW10, GW12, GW16, and GW23) from a total of 19 human embryos. After quality control and bioinformatics clustering analysis, CD45^+^CD33^+^ myeloid cells (12,565 cells) were selected and labeled for further analysis. 20 evident clusters (C1–C20) of myeloid cells were defined by combining graph-based clustering with known marker genes. At the same time, according to gene expression characteristics, these subgroups were divided into four meta-categories: origin, proliferation, immune response, and neuronal genes enrichment.

**Figure 1.**
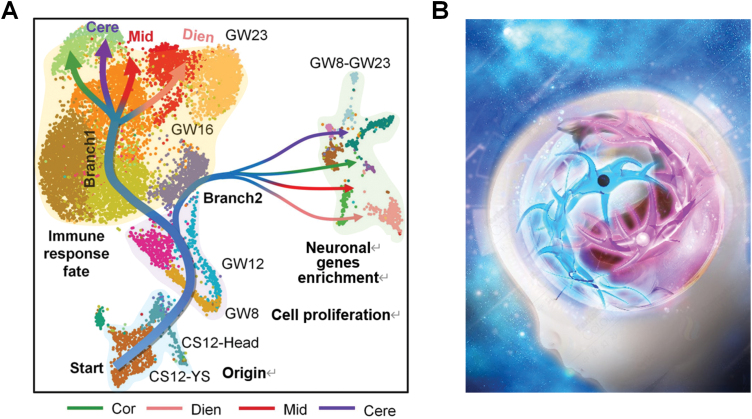
Microglia show the spatiotemporal dynamics of regional specification and state transition in the developing brain. (A) The schematic illustration reveals the developmental trajectories of immune-related and neuron gene-enriched microglia in distinct brain regions. (B) Tai Chi represents the simultaneous existence of immune-related and neuron gene-enriched microglia in the brain, which jointly maintain the corresponding network construction of each brain region.

To visualize and reshape the cell fate determination trajectories of neuron gene-enriched microglia and immune-related microglia from the same group of primordial myeloid precursor cells of CS12, they used Monocle2 and URD for pseudo time analysis. Interestingly, both analysis methods highlighted similar results: microglia progenitor cells derived from the CS12 yolk sac and head were the first to initiate cell proliferation, and then these proliferating microglia transited to two different cell fates: immune or neuronal gene enrichment characteristics (Fig. 1). Microglia transcriptional trajectories displayed a common intermediate stage for immune-related developmental branches and finally localized to a specific regional location in GW23. However, neuronal gene-enriched microglia with different regional characteristics come directly from circulating microglia. In addition, the study identified seven groups of microglia rich in neuronal gene modules and found that they all appeared instantaneously in four different regions including Cor, Dien, Mid, and Cere, and appeared briefly and dynamically to varying stages of early brain development as early as GW8. Homeostasis refers to a resting state of microglia, the study also focused on potential relationship between the regional specificity of fetal microglia and state transition. The authors propose that the regional specialization of immune-related microglia will be accompanied by the withdrawal of microglia from the resting state and further initiation of different immune activation states with regional specialization at GW23. The above findings outline the unique characteristics of fetal activation of immune-related microglia with regional specificity, different from all adult mature microglia data previously reported.

Furthermore, the authors compared the resting state transition of human and mouse microglia and found that fetal microglia’s dynamic resting/active state transition was conservative in humans and mice. After integration of human and mouse microglia data, the authors discovered that most other cell types can be combined, indicating that several cell types and changes of homeostasis-related genes of humans and mice are conserved, such as *CX3CR1*, *P2RY12,* and *TMEM119*. In addition, by analyzing the differentially expressed genes, they found some differences in the molecular characteristics of human and mouse microglia. For example, human microglia highly express *SPP1* and *C3*, while mouse microglia highly enriched genes of *atp6* and *cox2*.

In another paper published in Science [7], Kracht and colleagues studied the human fetal microglia in development and revealed the microglial homeostatic immune-sensing properties. Using a modified Smart-seq2 scRNA-seq, the authors profiled single-cell transcriptomics from 9 to 18 gestational weeks (GW9–18) of human fetal CNS tissue. Using fluorescence-activated cell sorting, human fetal microglia (CD11B^pos^CD45^int^) were isolated and sequenced (Altogether, 15,782 microglia cells). The authors found that microglia were heterogeneous at all GWs and exhibited an activated phagocytic profile. During brain development, microglia progressed toward more mature, immune-sensing competent phenotype, and increasingly resembled adult microglia with CNS-surveilling properties. This suggests that microglia might render the developing human CNS which is vulnerable to environmental perturbations during development, and as the CNS development progresses microglia endow a more homeostatic state, reflected by an increasing overlap between genes expressed in fetal, juvenile, and adult human microglia.

Taken together, these studies reveal the overall developmental trajectories of human embryonic microglia from the yolk sac to the brain, heterogeneity, the unique characteristics of fetal microglia state transition, and the close relationship between state transition and regional specificity during early brain development. These findings provide a basis for exploring the impact and pathogenic mechanism of microglia developmental deficits in early brain development on neuropsychiatric diseases such as autism, schizophrenia, and depression, and lay a foundation for further screening of therapeutic targets points. In addition, in the future, related transgenic methods can be used to change the related functions of human ES derived microglia cells, and the microglia cells can be purposefully modified, thereby providing therapeutic microglia cells for the treatment of related brain diseases.
